# Nonlinear dynamics in phosphoinositide metabolism

**DOI:** 10.1016/j.ceb.2024.102373

**Published:** 2024-05-25

**Authors:** Suet Yin Sarah Fung, X. J. Xǔ, Min Wu

**Affiliations:** 1 Department of Cell Biology, Yale University School of Medicine, 333 Cedar Street, New Haven, CT, 06520-8002, USA; 2 Department of Physics, Yale University, New Haven, CT, 06511, USA

## Abstract

Phosphoinositides broadly impact membrane dynamics, signal transduction and cellular physiology. The orchestration of signaling complexity by this seemingly simple metabolic pathway remains an open question. It is increasingly evident that comprehending the complexity of the phosphoinositides metabolic network requires a systems view based on nonlinear dynamics, where the products of metabolism can either positively or negatively modulate enzymatic function. These feedback and feedforward loops may be paradoxical, leading to counterintuitive effects. In this review, we introduce the framework of nonlinear dynamics, emphasizing distinct dynamical regimes such as the excitable state, oscillations, and mixed-mode oscillations—all of which have been experimentally observed in phosphoinositide metabolisms. We delve into how these dynamical behaviors arise from one or multiple network motifs, including positive and negative feedback loops, coherent and incoherent feedforward loops. We explore the current understanding of the molecular circuits responsible for these behaviors. While mapping these circuits presents both conceptual and experimental challenges, redefining cellular behavior based on dynamical state, lipid fluxes, time delay, and network topology is likely essential for a comprehensive understanding of this fundamental metabolic network.

## Introduction

**Figure 1:**
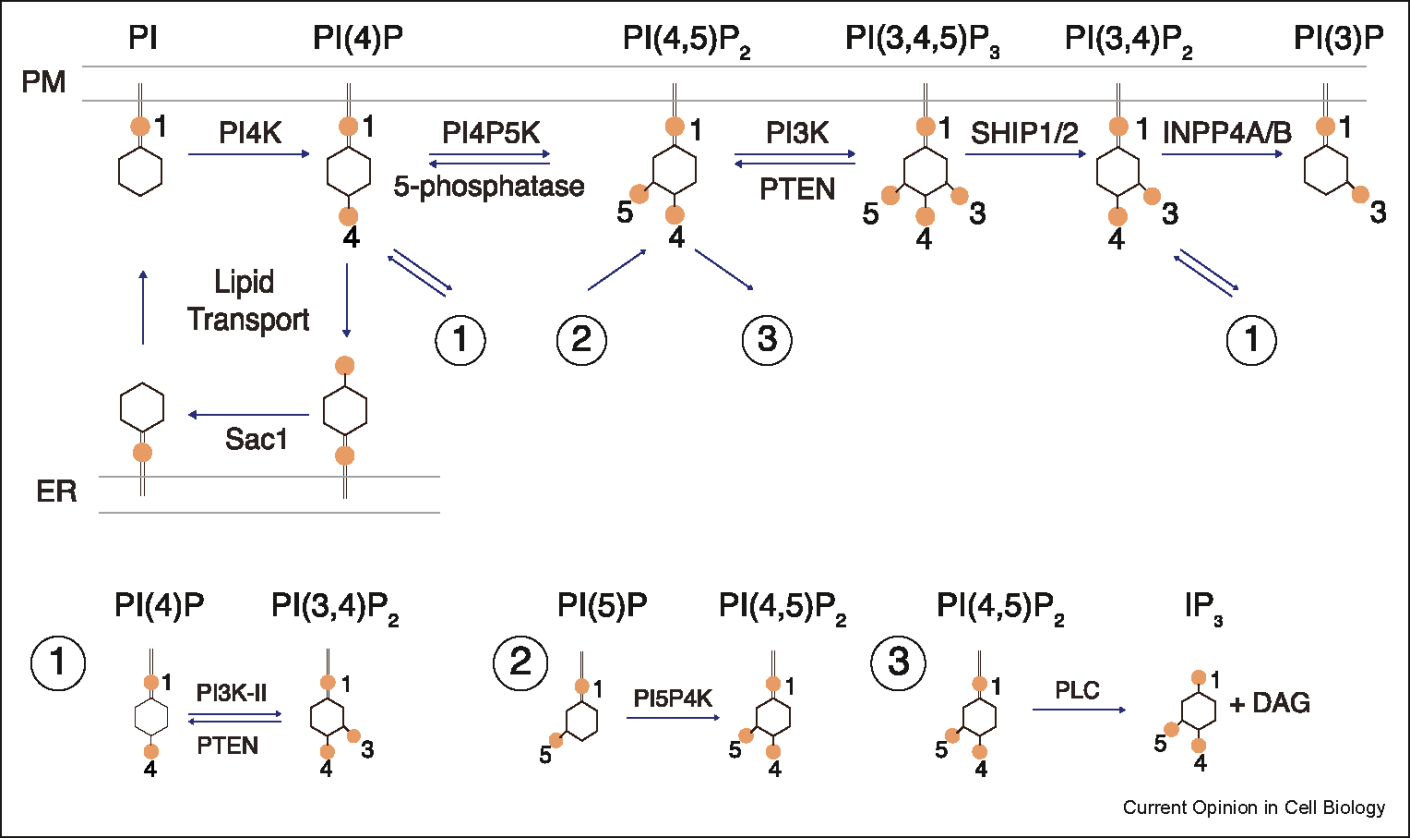
Phosphoinositides metabolism cascade and network. The synthesis of PI(4,5)P_2_ on the plasma membrane involves adding a 4-phosphate to Phosphatidylinositol (PtdIns or PI), generating phosphatidylinositol 4-phosphate (PtdIns(4)P or PI(4)P), followed by further addition of a 5-phosphate to produce PI(4,5)P_2_. PI(4,5)P_2_ can be further converted to phosphatidylinositol-(3,4,5) trisphosphate (PI(3,4,5)P_3_), phosphatidylinositol 3,4-bisphosphate (PtdIns(3,4)P_2_ or PI(3,4)P_2_), and phosphatidylinositol 3–phosphate (PI(3)P). This main cascade is accompanied by additional branches of metabolic reactions, including lipid transport of PI and PI(4)P between the ER and PM, direct conversion between PI(4)P and PI(3,4)P_2_ (1), synthesis of PI(4,5)P_2_ from a minor species PI(5)P (2), the hydrolysis of PI(4,5)P_2_ by phospholipase C to generate diacylglycerol in the plasma membrane and cytoplasmic inositol trisphosphate (Ins(1,4,5)P_3_) (3).

Phosphoinositides are low-abundance and transient signaling molecules that exert a ubiquitous impact on cell physiology by regulating membrane composition, organization and dynamics [1–5]. They exist in multiple species, each characterized by the phosphorylation status of the inositol ring. The conversion between these species involves the addition or removal of phosphate groups at different positions on the inositol ring by lipid kinases and phosphatases. Here we focus on plasma membrane (PM) phosphoinositides (Figure 1). This extensive metabolic network, centering around Phosphatidylinositol 4,5-bisphosphate (PtdIns(4,5)P_2_ or PI(4,5)P_2_), regulates receptor and ion channel activation, endocytosis, phagocytosis, exocytosis, virus budding and fusion, cytoskeletal dynamics and impacts almost all aspects of signaling from the plasma membrane, which in turn regulate diverse cellular functions such as nutrition and environment sensing, chemotaxis, immunity, development and neuronal activity.

## Nonlinear effects in phosphoinositides metabolism

While reactions mediating the conversions of phosphoinositides and pathways linking these enzymatic reactions seem well established, many counter-intuitive results have emerged in the recent decade with the development of precise methods for genetically and optogenetically perturbing specific enzymes. Instead of causing corresponding changes in the downstream events when an upstream node is perturbed, as one would predict from a linear cascade of metabolic reactions, these findings collectively challenge the linear interpretation of nearly every single step in this network. It has been consistently reported that inhibition of PI4-kinase (PI4K) III alpha, the major enzyme responsible for the synthesis of PI(4)P and PI(4,5)P_2_ on the plasma membrane, alters PI(4)P, but has either no [6,7] or minimal [8] impact on PI(4,5)P_2_. Loss of phosphatidylinositol-5-phosphate 4-kinases (PI(5) P4K), a minor pathway that synthesis PI(4,5)P_2_ from PI(5)P, in fact lead to an increase in PI(4,5)P_2_ and insulin-stimulated production of PI(3,4,5)P_3_ in Hela cells, B cell and drosophila cells [9–11]. Reports also indicate that reducing PI(4,5)P_2_, using a chemically inducible dimerization system, results in activation of PI(3,4,5)P_3_ and hyperprotrusive behavior at the tips of newly formed protrusions in *Dictyostelium* [12]. Similar results were also found in PC12 cells, where reducing PI(4,5)P_2_ by knocking down PIP5Ka potentiated NGF induced PI3K/Akt activation and neurite outgrowth, while exogenously applied PI(4,5)P_2_ to the knockdown cells suppressed Akt hyperactivation [13]. Furthermore, despite PI(3,4,5)P_3_ being a major precursor to PI(3,4) P_2_, recent experiments have shown that inhibition of PI(3,4,5)P_3_ could increase PI(3,4)P_2_ in *Dictyostelium* [14]. Similar paradoxical result was also noted in *Salmonella* infected host cells, which lead to a provocative model where PI(3,4)P_2_ is synthesized from PI(4,5)P_2_ via a phosphotransferase/phosphoisomerase mechanism, independently of phosphoinositide 3-kinases [15].

Lastly, the role of lipid transfer proteins poses an equally puzzling challenge. PI(4)P is synthesized on the PM by PI4-kinase (PI4KII alpha) but is degraded on the ER by Sac1 (the major lipid phosphatase involved in PI(4)P degradation) on the ER [16]. Consequently, both metabolism and lipid transport are crucial for controlling PI(4)P flux on the PM. Previous studies in yeast have shown that deletion of ER/PM tethering proteins leads to an increased PM PI(4)P [17,18]. However, such an effect requires the deletion of six yeast ER/PM tethering proteins, including three E-Syts. In HeLa cells, knockout of all three E-Syts did not result in differences in phosphoinositide levels [19]. These findings raise questions about the importance of these proteins for lipid transfer or whether their function is compensated by alternative lipid transfer proteins due to redundancies. Similar puzzles apply to other lipid transfer proteins, as detectable changes in lipid levels are rarely observed. When genetic perturbation was introduced and lipids changes were detected (sometimes involve deletion of multiple genes encoding lipid transfer proteins), they appear more like the exception than the rule [17,18,20].

## Logic underlying the linear intuition

Before delving into the necessity of understanding lipid metabolism through a nonlinear circuit perspective, it is crucial to reflect why the aforementioned results seem surprising and what aspects of the signaling might genuinely exhibit linearity. Although this review emphasizes the limitations of such linear interpretation, a linear approximation, applied with caution, remains necessary to simplify the complex network in a manner that is ultimately understandable.

Our inclination towards linear logic likely stems from several decades of reductionist thinking. Our linear intuition may originate from our understanding of metabolism at the single reaction level as well as at the network level. At the level of single reaction, most enzymatic activities characterized in vitro exhibit strong dose-dependent responses [21–24]. Although it is also widely acknowledged that enzymatic rates may exhibit non-linear dependencies on substrate concentration, attributed to factors such as Michaelis–Menten kinetics, positive cooperativity, or ultrasensitivity, this expectation arises from the dose–response curves depicting how product changes with enzyme concentration. Consequently, it is anticipated that reducing the enzymes responsible for synthesizing PI(4,5)P_2_ will lead to a reduction in PI(4,5)P_2_ levels. Similarly, a decrease in the PI(4)P lipid transfer protein in cells would be expected to result in a corresponding increase in PI(4)P levels. Translating such dose–response relationships from in vitro studies to living cells requires the assumption that in vivo concentrations lie within the dose-responsive range.

While deviation from linear behavior or dose-responsive curve could indeed occur at the level of individual reactions, such sources of nonlinearity alone are unlikely to account for completely opposite trends one sometimes sees. To address the more paradoxical results, it is imperative to critically evaluate the assumption that when multiple metabolic reactions are linked to form cascades of lipid metabolism pathways or networks, specific enzymes still remain rate-limiting, similar to the isolated reaction in vitro. At the network level, our intuitive feeling of a linear response likely arises from the fact that there is an overall progression along these cascades that is sequential and directional. The directionality of this cascade, which means no perturbation would lead to an opposite net flux from PI(3,4,5)P_3_ to PI(4,5)P_2_ and then PI(4)P, is remarkable. This is noteworthy considering that many of these metabolic steps are reversible due to the presence of lipid phosphatases, and maintaining this cascade consumes energy. However, the notion of rate-limiting enzymes can be challenged in various ways.

Firstly, the percentage of fluxes transduced from the first reaction to the next, when multiple metabolic steps are connected in sequence, is unknown. This becomes particularly challenging for reaction branches where the distribution among these branched pathways remains unclear. For example, if PI(4,5)P_2_ can be both degraded by phospholipase C (PLC) or metabolized to form PI(3,4,5)P_3_, the mechanisms controlling this partition are not well understood. Similarly, the competition of PI(4)P between its lipid transport/degradation path and the metabolic path to generate PI(4,5)P_2_ can introduce nonlinear effects.

**Figure 2:**
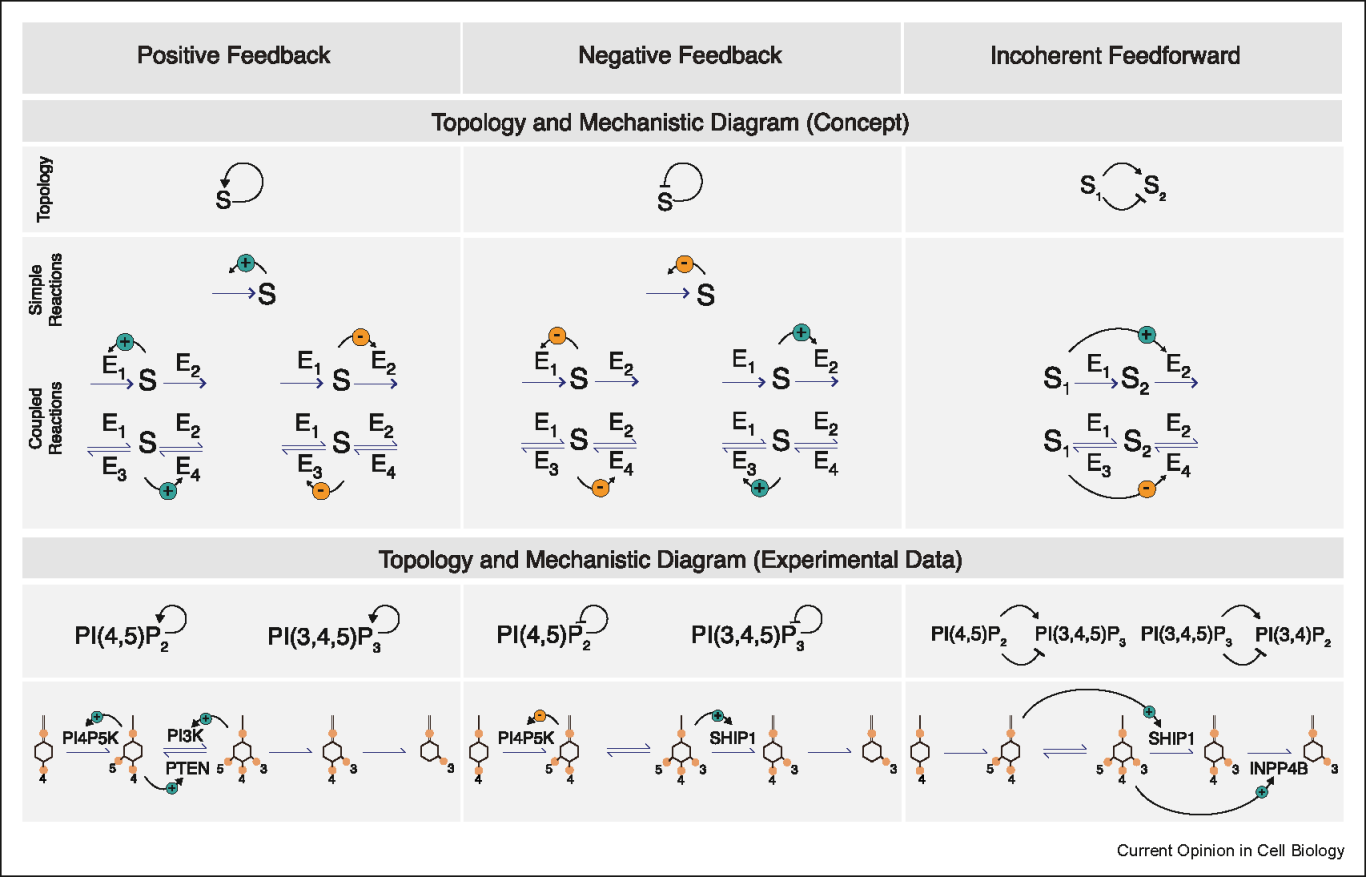
Topological and mechanistic diagram of the nonlinear feedback and feedforward regulations in phosphoinositides metabolism network.

Secondly, even in a linear cascade of enzymatic reactions, the reaction product can provide feedback or feedforward control over its upstream or downstream enzymatic steps, respectively. Here we employ two types of schematics: topological and mechanistic diagram to illustrate these non-linear effects (Figure 2). The topological diagram is an abstraction that focus on the positive or negative relation on the information flow between phosphoinositides, while the mechanistic diagram incorporates information on the chemical reaction schemes, i.e. how the substrate affects enzyme function. Generally, feedback regulation encompasses self-enhancement (positive feedback) and self-inhibition (negative feedback), while feedforward regulation can be a coherent or incoherent type, depending on whether the regulation exerts similar or opposite effect relative to what is expected from a linear cascade.

When multiple metabolic steps are connected, the number of mechanistically distinguishable circuits leading to the same type of topology increases. When two reactions are coupled in a sequence, both activating production and inhibiting degradation are topologically equivalent to positive feedback, while either activating inhibition or inhibiting the activator forms negative feedback (Figure 2). If the two coupled reactions are reversible, four distinct mechanistic circuits would correspond to each topology, respectively. These nonlinearity extend beyond the immediate upstream and downstream reaction. For example, if a substrate (S1) activates the enzyme (E2) that consumes the next substrate in the cascade (S2), it could create an incoherent feedforward loop, because S1 exerts both a positive effect on S2 (as a substrate for S2) and a negative effect (activating the degradation of S2) (Figure 2). Similarly, if a substrate (S1) inhibits the enzyme that produces the next substrate in the cascade (E4), it also forms an incoherent feedforward loop because S1 exerts both a positive and negative effect on S2 (Figure 2).

## Feedback and feedforward loops in phosphoinositides metabolism

How the metabolic diagrams are traditionally drawn only illustrate the possible reactions but fail to incorporate any non-linear effects, so they tend to mask the fact that the real information flow in the metabolic circuit remain largely unknown. Here we use the topological and mechanistic diagrams to summarize the current knowledge of non-linear feedback and feedforward loops in phosphoinositides metabolism obtained from experimental data.

### Positive feedback

For a cascade of PI(4)P ⇌ PI(4,5)P_2_ ⇌ PI(3,4,5)P_3_, a simple topology corresponding to the positive feedback of PI(4,5)P_2_ could theoretically originate from four distinct molecular circuits: activation of PI5K by PI(4,5) P_2_, inhibition of PI3K by PI(4,5)P_2_, activation of PTEN by PI(4,5)P_2_, or inhibition of 5^0^-phosphatase by PI(4,5) P_2_ (Figure 2). Some of these scenarios have been demonstrated experimentally. For examples, PI(4,5)P_2_ can recruit PTEN, which converts PI(3,4,5)P_3_ into PI(4,5)P_2_, leading to a positive feedback loop (PI(4,5) P_2_ →PTEN →PI(4,5)P_2_) [25,26]; Based on in vitro reconstitution on supported lipid bilayers, it was also found that PI(4,5)P_2_ can recruit PIP5KIa, forming a positive feedback loop (PI(4,5)P_2_ →PIP5Kia →PI(4,5)P_2_) [23,27]. Positive feedback of PI(3,4,5)P_3_ could involve PI(3,4,5)P_3_ and Rho GTPases [28–35]. For PI(3,4)P_2_, a mutually inhibitory loop between Ras and PI(3,4)P_2_ have been proposed to form positive feedback loops [36].

### Negative feedback

Negative feedback loops regulating PI(4,5)P_2_ and PI(3,4,5)P_3_ have both been reported (Figure 2). PI(4,5) P_2_-dependent recruitment of PIP4K, which synthesize PI(4,5)P_2_ via a minor pathway, could inhibit the major PI(4,5)P_2_-synthesizing PIP5Ks, forming a negative feedback loop (PI(4,5)P_2_ →PIP4K ⊣ PIP5K →PI(4,5) P_2_) [37]. A time-delayed negative feedback loop of PI(3,4,5)P_3_ (PI(3,4,5)P_3_ →Cdc42→FBP17 →SHIP1 ⊣ PI(3,4,5)P_3_) was proposed to regulate frequency of cortical Cdc42 oscillation in mast cells [38]. A similar negative feedback loop (PI(3,4,5)P_3_ →Rac/Cdc42 →SHIP2 ⊣ PI(3,4,5)P_3_) was also proposed for PC12 cells [39].

### Incoherent feedforward

In mast cell, a PI(4,5)P_2_-dependent incoherent feedforward loop overlaps with the PI(3,4,5)P_3_-dependent negative feedback loops that regulates Cdc42 activity (PI(4,5)P_2_→ PI(3,4,5)P_3_, with delayed feedforward inhibition PI(4,5)P_2_ ⊣ PI(3,4,5)P_3_, via PI(4,5)P_2_ →FBP17 →SHIP1 ⊣ PI(3,4,5)P_3_) [38] (Figure 2). These circuits regulate the activation cycle of Cdc42 and curvature-generating protein FBP17, which are sensitive to both PI(4,5)P_2_ and PI(3,4,5)P_3_. An incoherent feedforward loop was also suggested to regulate Rho activation (PI(3,4,5)P_3_ →PI(3,4)P_2_, PI(3,4,5)P_3_ →INPP4 ⊣ PI(3,4)P_2_) [40] (Figure 2).

## A dynamical systems framework

**Figure 3:**
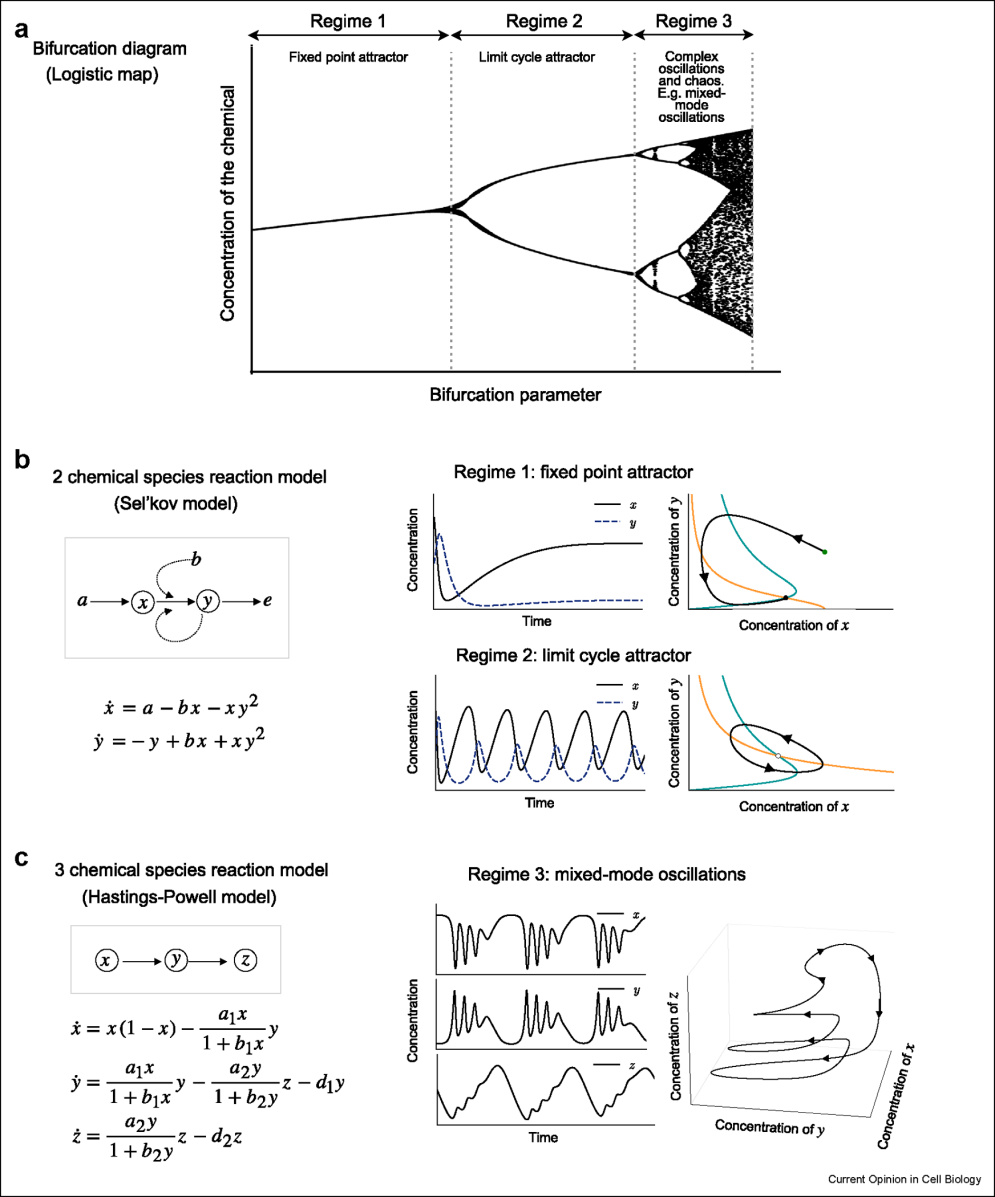
Minimal models illustrating distinct dynamical regimes. (**a**) A bifurcation diagram of the logistic map illustrates the conceptual connection between the 3 different regimes of dynamics. (**b**) The Sel’kov model for glycolysis involves 2 chemical species and it describes the dynamics of adenosine diphosphate (ADP) and fructose-6-phosphate (F6P) represented by x and y respectively. This system demonstrates excitable behavior as well as limit cycle oscillations of the substrate-depletion kind. (**c**) The 3 species Hastings–Powell model shows how the addition of a third state variables is necessary to generate far more complex behaviors, such as mixed-mode oscillations.

To integrate the impact of the above-mentioned nonlinear effects of lipid metabolism into functional circuits, additional information on the time scale and relative timing (phases) of all the reactions is essential. Although significant knowledge has accumulated on the biochemistry and genetics of the phosphoinositides metabolic enzymes, much less is known about the kinetics of these reactions in physiological contexts [41]. In principle, the interplay between network topology and kinetic (time scale and time delay) is where the complexity lies. Because the framework of dynamical systems have been extensively theorized in conceptually related fields, such as predator-prey relationships in ecology [42–44], nonequilibrium chemical reactions in physical chemistry [45–47], glycolysis in metabolism [48,49], we introduce the minimal models developed in these fields to illustrate how network topology together with kinetics can gives rise to these distinct dynamical regimes (excitable, simple oscillations and complex oscillations) (Figure 3a), and how these dynamical regimes can be used to maintain homeostasis, or govern temporal dynamics. The different regimes of dynamics and their transitions can be illustrated by a bifurcation diagram, which tells us how the dynamical behavior of the system changes as we change a single bifurcation parameter [50]. While these concepts have only been sparsely applied to phosphoinositides metabolism, recent experimental evidence suggests that similar complexities could readily emerge from the phosphoinositides network.

### Dynamical regimes 1: excitable dynamics

The first dynamical regime is excitable dynamics (Figure 3b top right panel). Understanding excitable dynamics and adaptation is essential for us to appreciate how the system can be perturbed (stimulated or inhibited) but returns to the same baseline, a phenomenon known as homeostasis [51–54]. Frequently, activation of lipid synthesis results in a transient increase in lipids (changes in the balances of lipid influx and efflux), lasting only a few seconds or minutes before it is degraded and returns to baseline. The majority of literature related to phosphoinositides metabolism can be classified in this regime, even though how the system was perturbed and how the following changes were measured differ. Apart from variations in ligand activation of surface receptors that stimulate phosphoinositide production and turnover, other important line of research involves processes such as endocytosis [55], macropinocytosis [14,56,57], phagocytosis [3,58], focal adhesion [59], and immune synapse [60]. Additionally, with the development of chemical dimerization and optogenetic methods of acutely inducing relocalization of lipid enzymes, the synthesis and degradation of lipids can be studied with unprecedented time resolution [61–64]. Regardless of the type of trigger, the overarching concept remains consistent: an eventual return to the baseline following a pulse of activation.

To understand excitable dynamics, we employ the Sel’kov model to introduce phase plane analysis (Box 1). We use a simplified form of the Sel’kov model [65], originally inspired by the study of glycolysis [49], to highlight the essential features of the system, namely an autocatalytic reaction where an enzyme activated by its product. We opt for the Sel’kov model over the more commonly used FitzHugh-Nagumo (FHN) model to explain excitability because the parameters in the FHN model lack biological meaning [49,66,67]. Using this simple model, one can use two ordinary differential equations to describe how reactant X and product Y change as a function of time, as well as a phase plane to visualize the relation between reactant X and product Y. With proper choice of parameter, the system could return to the same steady state regardless of the perturbation or initial conditions. This regime is referred to as the ‘Fixed Point Attractor’ because the steady state represents a fixed point in the phase plane, which is the homeostatic state of the system. The phase plane analysis can also mitigate the common confusion between the steady state and the flux because the amplitude and duration of the flux can vary depending on the trajectory which is different from where the fixed point is.

### Dynamical regime 2: oscillations

To understand the effect of network topology and kinetics, we next use the same reaction schemes of the Sel’kov model, but adjust a single parameter that corresponds to changing the flux rate. Now, the position of nullcline, equilibrium point and the overall vector field changes so that the equilibrium point is no longer stable. The system instead converges unto an isolated closed orbit in the phase plane, referred to as a limit cycle (Box 1). This is the second dynamical regime, characterized by self-sustained oscillations (Figure 3b lower right panel). When we compare the excitable and oscillation regimes, two key concepts can be illustrated. Firstly, network topology is critical in predicting whether the system will return to homeostasis. Whether the steady state for each regime is a stable fixed point or limit cycle in the phase plane does not depend on the initial concentrations but relies on the sign of interactions (topology) and kinetic rates (Box 1). Secondly, the same set of metabolic reactions can readily give rises to distinct network topologies with small changes of kinetic parameters, or flux rates.

It is natural to wonder whether there are general principles regarding the network topology that can predict the stability of the system, which is a subject that has been extensively discussed in previous reviews on dynamical systems in biology [68–70], chemistry [47,71] and the classic metabolic flux control theory [72–74]. Briefly, it turns out that for two component system, there are only two topologies that can give rise to oscillations, commonly known as the substrate-depletion model and the activator-delayed inhibitor model [75]. The Sel’kov model can permit oscillations of the substrate-depletion topology. The Gierer-Meinhardt model, on the other hand, displays self-sustained oscillations of the activator-inhibitor topology [76].

In recent years, advancements in biosensors and imaging techniques have enabled the observation of phosphoinositide oscillations in various single-cell systems. Strictly speaking, biosensors measure accessible pool of the lipids, rather than the absolute concentration of total lipids, but the fluctuation of free lipids level (as seen for a given binding affinity) is likely what is physiologically relevant for dynamically recruiting cytoplasmic proteins to the membrane. Notably, PI(3,4,5)P_3_ oscillations in *Dictyostelium* [12,25,77–80], antigen-stimulated mast cell [38], and glucose-stimulated beta-cell [81–83]. Additionally, PI(4,5)P_2_ oscillations have been documented in mast cell [38,40,84–86] and neurons [87], while PI(3,4)P_2_ oscillations have been observed in mast cells [38,88].

Both network topologies capable of generating oscillations have been observed for PI(4,5)P_2_-PI(3,4,5)P_3_ oscillations. In mast cell, cortical PI(4,5)P_2_ and PI(3,4,5)P_3_ oscillations follow the activator-delayed inhibitor model. These oscillations are coupled with SHIP1 and PI(3,4)P_2_ oscillations, as well as cycles of Cdc42 activation [38] and actin turnover [89]. PI(4,5)P_2_ was not depleted but rise and drop together with PI(3,4,5)P_3_. Lipid phosphatase SHIP1 act as delayed inhibitor [38]. In contrast, the propagation of PI(3,4,5) P_3_ waves have also been observed to be accompanied by the consumption of its substrate PI(4,5)P_2_, consistent with the substrate-depletion model, including oscillations of PI(4,5)P_2_ depletion linked to Ras oscillations in *Dictyostelium* [25,77,78,90] and nocodazole-induced Rho oscillations in mast cell [40]. It may not be immediately apparent how PI(3,4,5)P_3_ can be generated while the PI(4,5)P_2_ level is depleted. This is explained by the presence of a basal level of PI(4,5)P_2_ to start with, and the depletion occurs due to the net efflux compared to influxes, causing a reduction in the accessible pool of free PI(4,5)P_2_.

If the same reaction scheme responsible for excitable dynamics can also induce oscillation with simple changes of flux rate, understanding the basic form of the oscillation, i.e., the limit cycle attractor, would be a better alternative towards understanding excitable dynamics or homeostasis. Dissecting the molecular circuit that establishes the excitable and adaptation of lipid homeostasis is informative [91]. However, with increasing number of molecular circuits identified, many single layer feedback mechanisms appear to be redundant. Notably, while there is compelling evidence that PI(3,4,5)P_3_ defines the cell front and excitability, there are genetic evidence that PI(3,4,5)P_3_ is not essential for excitability in cell migration [92]. A number of alternative circuits have been proposed [36,93]. Such apparent redundancy or degeneracy poses the same conceptual challenges facing the mapping of neural circuits where multiple solutions produce similar outputs [94]. In contrast, oscillations offer temporal information on the refractory phase that is invisible from an excitable state (unless repetitive stimuli are introduced to an excitable state), providing additional constraints on the inhibitory arm of the circuit. In doing so, dissecting networks driving oscillations can reveal inhibitors that differentially regulate the duration or the refractory phase but appear redundant when just mapping molecular circuits in an excitable state.

### Dynamical regime 3: complex oscillations

The third dynamical regime is characterized by more complex oscillatory dynamics such as mixed-mode oscillations (Figure 3c). Mixed-mode oscillations are conceptually interesting for at least two reasons. First, the complexity of signaling networks is unlikely explained by a single limit cycle that acts like a master clock. Mixed-mode oscillations arise through coupling of two circuits operating on different time scales, so it contains additional information on the higher-level network that is missing in the regime supporting simple oscillations. Secondly, mixed-mode oscillations could offer distinct mechanism to explain how a system can shift states from one attractor to the next. The Hastings–Powell model is a minimal model to illustrate that the coupling of reactions in series can lead to a significantly expanded range of dynamic behavior. This ranges from simple limit-cycle oscillations to period-doubling or even deterministic chaos [95]. The mathematical essence lies in the network regulated by the coupled fast and slow circuits, where the slower reaction functions as a time-dependent variable that controls the flux for the fast circuit and determines whether it will oscillate or not.

Complex oscillations of phosphoinositides have recently been described in the context of contractility pulses [40]. These cycles of Rho activations exhibit heterogeneity in their oscillation periods as well as mixed-mode frequencies. The fast circuit involves the incoherent feedforward loop (PI(3,4,5)P_3_ →INPP4 ⊣ PI(3,4)P_2_), oscillating with a period of about 20 s. This loop suggests that PI(3,4)P_2_ serves as an inhibitor for Rho activation, likely due to PI(3,4)P_2_-dependent recruitment of RhoGAPs [59]. The slow circuit (operating over minutes) involves a PI(4)P-PI(4,5)P_2_ oscillator following the substrate-depletion model. This loop likely includes strong positive feedback of PI(4,5)P_2_ that depletes PI(4)P. The dynamical regime between simple and complex oscillations is controlled by the lipid transfer protein E-Syt1.

The higher-dimensional network view offered by complex oscillations is likely relevant for understanding seemingly simple oscillatory dynamics such as calcium oscillations as well. We primarily focus on PI(4,5)P_2_ oscillations that are not coupled with Ca^2+^/PLC/diacylglycerol (DAG) oscillations because considering Ca^2+^-dependent feedback introduces an additional level of complexity that currently lacks a consensus view. In classic literature, PI(4,5)P_2_/IP_3_ oscillations are associated with calcium oscillations [96–98]. This historical correlation with calcium has biased the mechanistic understanding of PI(4,5)P_2_ oscillation in a Ca^2+^/PLC/IP_3_ pathway-centric way. However, recent work in mast cells suggests that PI(4,5)P_2_ oscillations can be uncoupled from calcium oscillation or PLC activation. The form that is coupled with Ca^2+^ oscillations and the PLC/DAG pathway appears as a standing wave [85], while the form that is not coupled with Ca^2+^ oscillation appears as travelling wave [38]. Presence of bursting and mixed-frequencies in calcium oscillations indicate Ca^2+^ dynamics is likely controlled by a higher-dimensional network rather than by a simple limit cycle [99–101].

## Outlook

We suggest that the dynamical framework is essential to understand the complexity and nonlinear effects observed in phosphoinositides metabolism. Our focus is on plasma membrane phosphoinositides due to the availability of high-resolution dynamical information that is more attainable on the PM. Given the simplicity of the network topology needed for generating these dynamical behaviors, it is reasonable to expect their prevalence. Indeed, PI(4)P oscillations at the Golgi [102] and rapid PI(3)P waves form on the ER induced by intracellular bacterial pathogen upon infection of the host cells [103]. Although oscillations of other phosphoinositides such as PI(3,5)P_2_ have not been observed in cells, the complex feedback regulation revealed in vitro [104] suggests that PI(3,5)P_2_ could readily oscillate in cells. Interestingly, while PI(3,5)P_2_ has been thought to be primarily on the lysosomes, it is recently implicated in plasma membrane signaling for root hair morphogenesis in *Arabidopsis* and for establishing the back state of *dictyostelium* [93,105].

Despite the central importance of lipid flux, directly quantifying these processes is challenging. Radio-labeling coupled to organelle fractionation suffers from poor temporal resolution and impurities inherent in ultracentrifugation-based fractionation methods. Several chemoenzymatic methods for measuring lipid transfer and flux (METALIC [106], IMPACT [107]) have been developed, with promising results revealing kinetic differences caused by lipid transfer proteins [107,108]. It is likely crucial to characterize metabolic rates between different systems. For example, recent work suggest that hippocampal neurons display a much faster PI(4,5)P_2_ resynthesis rate compared to astrocytes [109].

Accumulating evidence supports the idea that transient lipid flux significantly impacts signaling. While the system rapidly returns to the steady state to maintain lipid homeostasis after local and transient lipid flux, the impact of fluxes propagates through the signal transduction network like ripples. Over time, defects in cell physiology or disease states develop, while measurable differences in steady-state lipid levels remain subtle. Dysfunction of virtually all phosphoinositide-metabolizing enzymes has been linked to human diseases such as cancer and inflammation [4,110]. However, clinical success is rare. For heavily invested drug targets such as PI3K, drug treatment remains challenged by on-target adverse effects due to the ubiquitous presence of this pathway in all tissue types and the diverse processes it regulates [111]. Biologists have intuitively attributed some of the enigmatic results associated with perturbation of PI(4,5)P_2_ and PI(3,4,5)P_3_ metabolism to the “butterfly effect” [11,112]. However, many are unaware of the framework of dynamical systems that explain the butterfly effects (chaos), or oscillations. Recognizing and dissecting these nonlinear effects in these fundamental biochemical reactions will be essential for redefining cell physiology and pathology with a dynamical systems framework.

## Data Availability

No data was used for the research described in the article.

## Box 1. Phase plane and linear stability analysis to illustrate the concept of fixed-point attractor and oscillation (limit cycle attractor) as well as how small changes of kinetic parameters could alter the dynamical behavior of the system.

- Use the relevant biochemical reactions, their rate laws to write a corresponding system of ordinary differential equations. Upper case letters represent chemical concentrations and lower case letters represent the state variable. The nullclines are found by setting x· and y· to zero and balancing the equations.
- Plot the nullclines and their intersection, which is the fixed point. The fixed point may be stable or unstable, depending on the vector field but not the initial condition. We show how changing kinetic rate a from 0.2 to 0.6 will shift the shape of the trajectory. The same initial condition leads to either a fixed point or a closed orbitals in the phase plane (a limit cycle), which is equivalent to the oscillatory of x and y concentration.
- Find the fixed point for each dynamical regime.
- Carry out linear stability analysis about the fixed point in order the classify the type of dynamical behavior analytically. Define the Jacobian interaction matrix and calculate the respective eigenvalues for the stable spiral regime and limit cycle regime. The two sets of parameters used are *a* = 0.2, *b* = 0.1 and *a* = 0.6, *b* = 0.1 and for the stable node and stable limit cycle cases respectively. The crossing of the imaginary number line indicates a transition from a stable fixed point to an unstable spiral, which lies at the core of the stable limit cycle.
- The change in network topology is reflected in the corresponding sign change the Jacobian.
